# P16 and CD44 as Biomarkers for Predicting the Progression of Immature Polypoid Squamous Metaplasia of the Cervix

**DOI:** 10.7759/cureus.81661

**Published:** 2025-04-03

**Authors:** Tamar Svanadze, Teona Turashvili, Shota Kepuladze, George Burkadze

**Affiliations:** 1 Obstetrics and Gynecology, Tbilisi State Medical University, Tbilisi, GEO; 2 Pathology and Oncology, Tbilisi State Medical University, Tbilisi, GEO; 3 Molecular Pathology, Tbilisi State Medical University, Tbilisi, GEO

**Keywords:** biomarkers, cd44, cervical intraepithelial neoplasia (cin), dysplasia progression, hpv, immature polypoid squamous metaplasia (ipm), p16

## Abstract

Cervical intraepithelial neoplasia (CIN) is a well-established precursor of cervical cancer, while immature polypoid squamous metaplasia (IPM) has been hypothesized as a possible early lesion in cervical carcinogenesis. However, the mechanisms underlying IPM progression to CIN remain unclear. Therefore, identifying reliable biomarkers to predict progression is crucial. This study evaluates the immunohistochemical expression of P16, CD44, estrogen receptor (ER), and progesterone receptor (PR) in CIN and IPM, focusing on their prognostic significance in IPM-to-CIN progression.

A total of 227 cervical tissue samples were analyzed, including CIN1 (N=30), CIN2 (N=30), CIN3 (N=32), IPM (N=60), mature squamous metaplasia (N=45), and reactive ectocervix - control (N=30). P16, CD44, ER, and PR expression were assessed immunohistochemically. Additionally, clinical HPV (human papillomavirus) status was determined via PCR, and marker expression was correlated with lesion grade using a standardized study algorithm. A subgroup of 40 IPM cases that progressed to CIN was analyzed to identify possible markers predictive of progression.

Our results demonstrate that higher P16 and CD44 expression levels are significantly associated with higher-grade CIN lesions (p < 0.001). P16 and CD44 expression also strongly predicted IPM progression: P16 showed a 100% positive predictive value (PPV) for progression to CIN2 or CIN3, meaning all IPM cases with moderate or strong P16 expression advanced to high-grade CIN. CD44 had a 90% PPV for CIN progression, suggesting a strong correlation with aggressive epithelial transformation.

Although ER and PR expressions were statistically significant (p < 0.05), they were not predictive markers of CIN progression. These findings highlight P16 and CD44 as possible biomarkers for identifying high-risk IPM lesions and assessing the likelihood of progression to CIN2/3.

## Introduction

Cervical intraepithelial neoplasia (CIN) refers to a spectrum of precancerous cellular changes in the cervix graded as mild dysplasia (CIN1), moderate dysplasia (CIN2), and severe dysplasia or carcinoma in situ (CIN3). CIN is a well-established precursor to cervical cancer, making timely detection and management crucial to prevent progression to invasive disease [1]. Current CIN diagnoses primarily rely on histopathological evaluation; however, variability in interpretation underscores the need for improved diagnostic markers. Human papillomavirus (HPV) infection is considered a primary etiological factor in the development of CIN; however, not all HPV infections result in the progression of CIN, and additional molecular factors play a role in this process [2].

Immature polypoid squamous metaplasia (IPM) is a distinct histological entity of the cervix characterized by the formation of gland-like, polypoid structures arising from squamous epithelium [3]. This condition has attracted increasing attention due to its potential role as an early morphologic change that may precede or coexist with CIN, particularly in individuals with persistent HPV infections. IPM frequently arises in the cervical transformation zone, the primary site for metaplastic changes and CIN development [4]. Although it has been considered a benign lesion, its association with HPV infection and the potential for progression to higher-grade dysplasia highlight the need for further investigation [5].

The histopathological features of IPM include epithelial cell hyperplasia, which may be associated with basal cell proliferation and alterations in the extracellular matrix, creating a more favorable environment for developing dysplastic lesions. Although it is not universally recognized as a precursor to CIN, emerging evidence suggests that IPM may represent an intermediate step in the pathway of cervical carcinogenesis, particularly when accompanied by HPV infection [6]. Molecular changes may facilitate the progressive transition from IPM to CIN, but the specific markers and mechanisms remain incomplete. Immunohistochemical (IHC) biomarkers have gained increasing importance in the diagnosing and prognosis of CIN and related lesions. The expression of p16, a cyclin-dependent kinase inhibitor, is widely utilized as a surrogate marker for high-risk HPV infection, and its upregulation is often associated with the presence of dysplasia in CIN [7]. Hormone receptors, particularly estrogen receptors (ERs) and progesterone receptors (PRs), play a role in regulating cervical epithelial homeostasis and modulating immune responses, which may influence the progression of CIN [8]. CD44, another promising marker, is a cell surface glycoprotein (tumor stem cell surface marker) involved in cell-cell interactions, differentiation, tumor cell migration, and signal transduction. Growing evidence supports its role in tumor progression, epithelial-mesenchymal transformation (EMT), and metastasis [9].

Despite the significant research on these markers, the precise relationship between P16, hormone receptors, and CD44 expression in the context of IPM and CIN has not been extensively studied. This study aimed to evaluate the IHC expression of P16, ER, PR, and CD44 in different stages of CIN and IPM to understand better the molecular changes involved in the progression of cervical lesions and to evaluate their potential as diagnostic and prognostic biomarkers. Analysis of these markers may reveal new insights into the process of cervical carcinogenesis, further emphasizing their clinical importance in improving early detection and management strategies.

## Materials and methods

Study design

Our study is a retrospective cohort observational analysis that evaluates the IHC expression of P16, ER, PR, and CD44 in cervical tissue samples indicative of different stages of CIN and IPM. The study received approval from the Ethics Review Board of Tbilisi State Medical University, and all data used were de-identified, anonymized, and kept confidential.

Sample selection

The cohort consisted of 227 formalin-fixed paraffin-embedded (FFPE) cervical tissue samples, comprising biopsies and hysterectomy specimens. These tissue samples were obtained from the Tbilisi State Medical University Pathology Teaching and Diagnostic Laboratory archives between 2017 and 2025.

The study cohort also included a subgroup of patients with a previous history of IPM who later progressed to CIN (n=20) (during 2017-2025). These cases were explicitly selected to evaluate potential markers associated with the progression of IPM to CIN.

The study included biopsy or hysterectomy specimens from patients diagnosed with CIN, IPM, or reactive changes and sufficient tissue for IHC analysis. Exclusion criteria included specimens with insufficient histological characteristics or poor tissue fixation and patients who had received HPV vaccination.

HPV status was determined using polymerase chain reaction (PCR) testing for high-risk HPV strains (e.g., HPV16, HPV18) in a subset of 150 cases based on patient clinical information. HPV testing was incorporated into the study algorithm to assess the association between HPV infection and expression of IHC markers at different stages of CIN and IPM. HPV positivity was categorized as positive or negative, and the correlation of high-risk HPV types with dysplastic lesions was determined.

Tissue processing and IHC studies

IHC Protocol

Tissue sections were deparaffinized in xylene and rehydrated with ethanol solutions. Antigen retrieval was performed using a citrate buffer (pH 6.0) and an adjustable device for 20 minutes. Endogenous peroxidase activity was blocked by incubating the slides with 3% hydrogen peroxide for 10 minutes. The slides were then incubated at 4°C with primary antibodies at optimized dilutions. Secondary antibodies conjugated to peroxidase (HRP) were used for 30 minutes at room temperature. Chromogenic detection was performed using 3,3’ diaminobenzidine (DAB).

Quality Control

Positive control refers to tissues known to express the target antigens, ensuring that the antibody staining and experimental conditions are functioning correctly. The following primary antibodies were used for IHC staining: P16 (clone E6H4, Roche Diagnostics, Basel, Switzerland) in a dilution of 1:100, ER (clone SP1, Leica, Buffalo Grove, IL) in a dilution of 1:200, PR (clone 16, Leica) in a dilution of 1:200, and CD44 (clone 156-3, Leica) in a dilution of 1:100.

After being incubated with primary antibodies, slides were incubated with secondary antibodies and then visualized using a DAB chromogen kit (Leica Novolink Polymer Detection System). They were then counterstained with hematoxylin, dehydrated, and mounted for microscopic examination.

IHC Staining Assessment

IHC expression of each marker (P16, ER, PR, and CD44) was assessed based on both the intensity and proportion of positive cells. A semiquantitative scoring system was used as follows:

P16: positive staining was defined as nuclear and/or cytoplasmic staining in ≥ 50% of epithelial cells. Intense staining was scored as 3+, moderate as 2+, weak as 1+, and no staining as 0.

ER and PR: nuclear staining in epithelial cells was assessed, with positivity defined as staining in ≥10% of cells. Staining was graded as absent (0), weak (1+), moderate (2+), and intense (3+).

CD44: membranous staining was assessed, with positivity defined as staining of ≥10% of epithelial cells. Staining was graded as weak (1+), moderate (2+), and intense (3+).

The study assessed marker expression at different stages of CIN and IPM and compared it in cases where IPM progressed to CIN. Particular attention was paid to identifying markers that could be prognostic indicators of IPM to higher-grade dysplasia or carcinoma.

Statistical analysis

Data analysis was performed using SPSS Statistics Version 30 (IBM Corp., Armonk, NY). Descriptive statistics were used to summarize the distribution of IHC expression across categories. The association between P16, ER, PR, and CD44 expression and histological diagnoses (CIN1, CIN2, CIN3, IPM, and control) was assessed using the chi-square tests or Fisher's exact test. A p-value of <0.05 was considered statistically significant. In addition, correlations between marker expressions were evaluated using Pearson's correlation coefficient. For the subset of cases where IPM progressed to CIN, longitudinal analyses were performed to assess the progression of marker expression and its potential association. Comparative analysis was performed to assess marker expression differences between progressive and non-progressive IPM cases to identify potential biomarkers of progression.

Ethical considerations

The study was conducted according to the Declaration of Helsinki and approved by the Institutional Review Board of Tbilisi State Medical University (N5-2020/82). All patient data were anonymized, and informed consent was withdrawn due to its retrospective nature.

## Results

Patient characteristics

A total of 227 cervical tissue samples, including biopsy and hysterectomy specimens, were studied in this study: CIN1 30 cases (13.2%), CIN2 30 cases (13.2%), CIN3 32 cases (14.1%), IPM 60 cases (26.4%), mature squamous metaplasia 45 cases (19.8%), reactive control 30 cases (13.2%).

The mean age of the patients was 40.6 years, with a range of 25 to 70 years. HPV status was determined for 150 cases, of which 120 (52.8% of total cases) cases were positive for high-risk HPV strains (HPV16, HPV18), and 30 (13.2%) cases were negative. A subgroup of 40 (17.6%) patients previously diagnosed with IPM who later progressed to CIN was analyzed separately to identify markers associated with progression from IPM to CIN.

Expression of P16, ER, PR, and CD44

**Figure 1:**
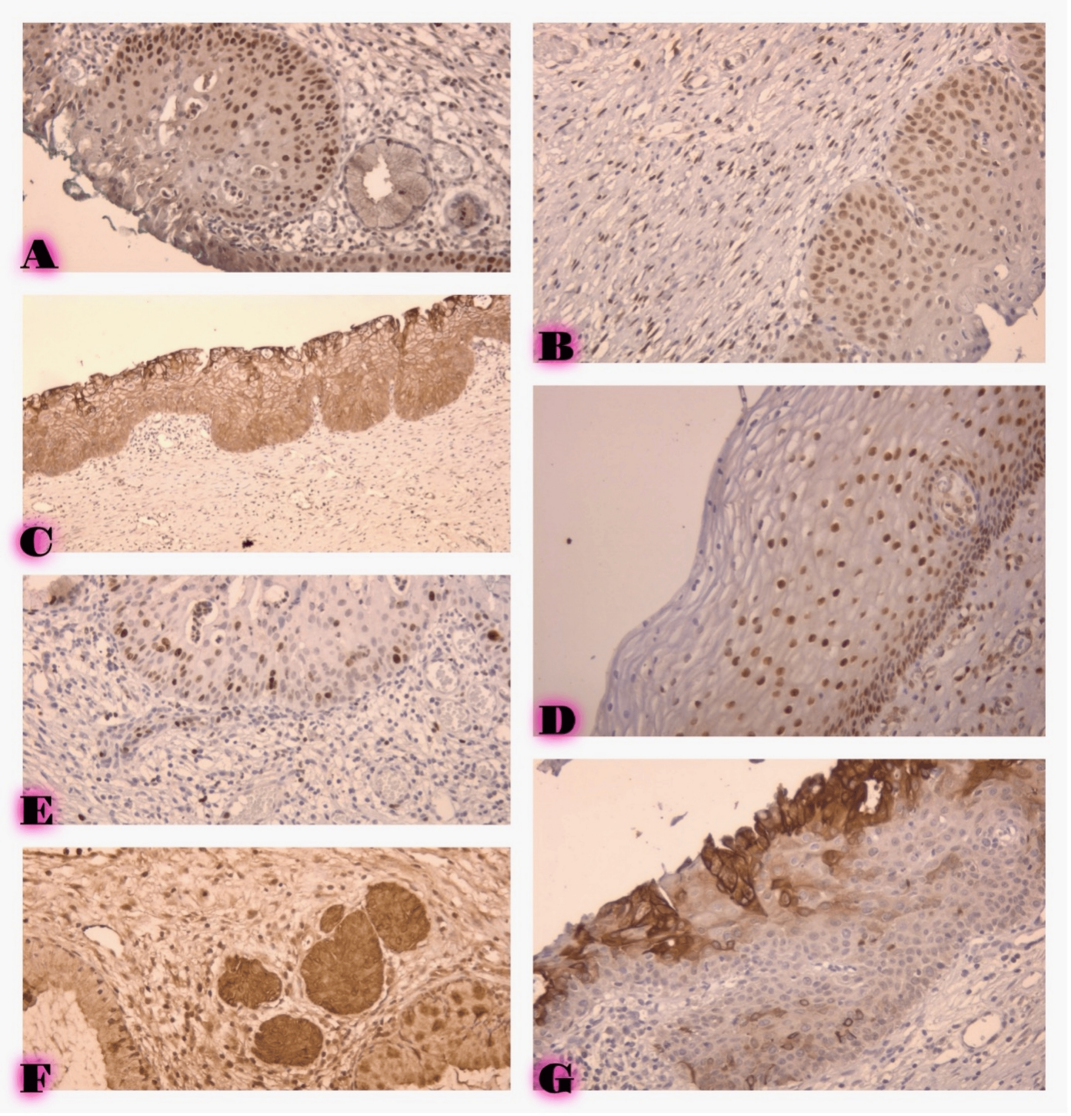
A. Immature squamous polypoid metaplasia ER expression, IHC, 400X. B. Low-grade cervical intraepithelial neoplasia (CIN1), ER expression, IHC, 400X. C. Low-grade cervical intraepithelial neoplasia (CIN1), CD44 expression, IHC, 400X. D. Low-grade cervical intraepithelial neoplasia (CIN1), PR expression, IHC, 400X. E. Immature squamous polypoid metaplasia P16 expression, IHC, 400X. F. Immature squamous polypoid metaplasia with low-intensity CD44 expression, IHC, 400X. G. Immature squamous polypoid metaplasia with high-intensity CD44 expression, IHC, 400X.

IHC expression of P16, ER, PR, and CD44 in each histological was assessed. The distribution and intensity of markers expression are summarized below, and visual manifestation is shown in Figure 1.

P16 Expression

CIN1: P16 expression was observed in 80% (N=24) of cases, with 70% (N=21) showing moderate expression (2+), 10% (N=3) showing strong expression (3+), and 20% (N=6) showing negative expression.

CIN2: P16 expression was observed in 100% (N=30) of cases, with 50% (N=15) showing weak expression (1+), 20% (N=6) showing moderate expression (2+), and 30% (N=9) showing strong expression (3+).

CIN3: P16 expression was observed in 100% (N=32) of cases, with 12.5% (N=4) showing weak expression (1+), 37.5% (N=12) showing moderate expression (2+), and 50% (N=16) showing strong expression (3+).

IPM: P16 expression was observed in 100% (N=60) of cases, with 50% (N=30) showing weak expression (1+), 50% (N=30) showing moderate expression (2+), and no strong (3+) cases.

Reactive control: No P16 expression was observed in 100% (N=30) of reactive control samples.

ER Expression

CIN1: ER expression was observed in 100% (N=30) of CIN1 cases, with 3% (N=1) showing weak staining (1+), 97% (N=29) showing moderate staining (2+), and no strong staining (3+).

CIN2: ER expression was observed in 90% (N=27) of CIN2 cases, with 70% (N=21) showing weak staining (1+), 20% (N=6) showing moderate staining (2+), and no strong staining (3+).

CIN3: ER expression was observed in 87.5% (N=28) of CIN3 cases, with 87.5% (N=28) showing weak staining (1+), no moderate (2+), and no strong staining (3+).

IPM: ER expression was observed in 100% (N=60) of IPM cases, with 7% (N=4) showing moderate staining (2+), 93% (N=56) showing strong staining (3+), and no weak staining (1+).

Reactive control: ER expression was observed in 100% (N=30) of reactive control cases, with all cases (100%) showing strong staining (3+), and no moderate (2+) or weak (1+) staining.

CD44 Expression

CIN1: CD44 expression was observed in 100% (N=30) of CIN1 cases, with 27% (N=8) showing moderate staining (2+), 73% (N=22) showing strong staining (3+), and no weak staining (1+).

CIN2: CD44 expression was observed in 100% (N=30) of CIN2 cases, with 10% (N=3) showing moderate staining (2+), 90% (N=27) showing strong staining (3+), and no weak staining (1+).

CIN3: CD44 expression was observed in 100% (N=32) of CIN3 cases, with 100% (N=32) showing strong staining (3+), no moderate (2+), and no weak (1+).

IPM: CD44 expression was observed in 100% (N=60) of IPM cases, with 100% (N=60) showing moderate staining (2+), and no weak (1+) or strong (3+) staining.

Reactive control: CD44 expression was observed in 100% (N=30) of reactive control cases, with all cases (100%) showing weak staining (1+), and no moderate (2+) or strong (3+) staining.

**Figure 2:**
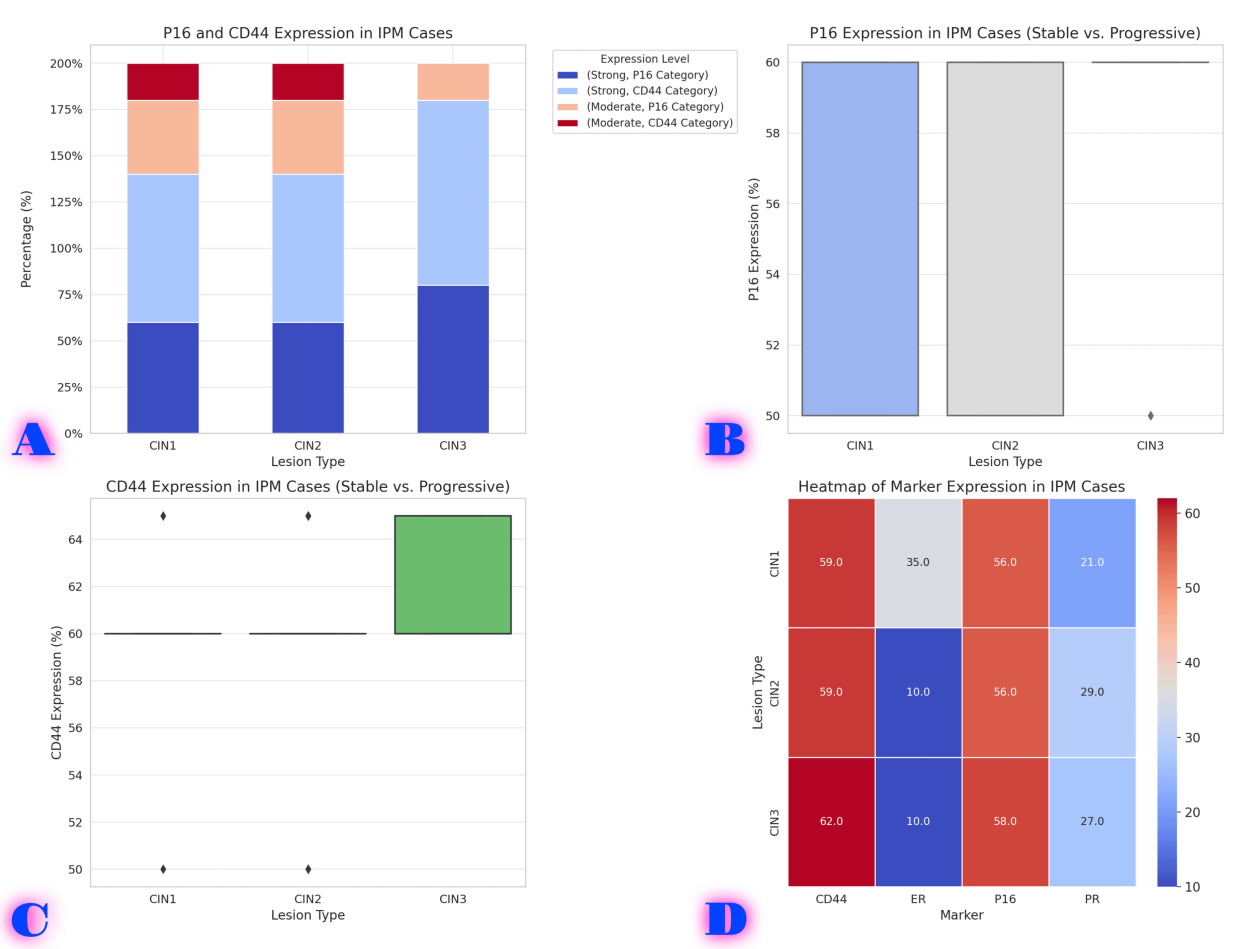
Biomarker expression patterns in IPM cases and their progression to CIN. A. Stacked bar chart showing P16 and CD44 expression levels in progressive vs. stable IPM cases. B. Boxplot (P16 expression) showing the comparison of P16 levels in stable vs. progressive IPM cases. C. Boxplot (CD44 expression) showing the comparison of CD44 levels in stable vs. progressive IPM cases. D. Heatmap showing the mean expression levels of P16, CD44, ER, and PR in different IPM cases. CIN, cervical intraepithelial neoplasia; IPM, immature polypoid squamous metaplasia

Among the 40 cases of IPM, all cases demonstrated progression to varying grades of CIN: 5 cases progressed to CIN1, 15 cases progressed to CIN2, 20 cases progressed to CIN3 (Figure 2).

P16 Expression

Overall, 100% (N=40) of progressive IPM cases exhibited moderate (2+) or strong (3+) P16 expression, reinforcing its role in lesion progression. No cases displayed weak (1+) P16 expression.

CD44 Expression

Overall, 91.4% (N=40) of progressive IPM cases demonstrated strong (3+) CD44 staining, indicating a potential role in tumor cell adhesion, invasion, and progression. These findings highlight P16 and CD44 as key biomarkers in the transition from IPM to CIN, particularly in high-grade lesions (CIN2/3). The significant difference in CD44 expression (p < 0.05) further suggests its potential as a predictive marker for disease progression.

Statistical analysis

Chi-square tests revealed a strong and statistically significant association between P16 expression and CIN severity (p < 0.001). Higher P16 expression correlated with more advanced CIN stages (CIN2 and CIN3), reinforcing its role as a key biomarker in disease progression. Similarly, CD44 expression was significantly higher in CIN2 and CIN3 compared to IPM and reactive controls (p < 0.001), suggesting its involvement in epithelial transformation and invasion.

**Figure 3:**
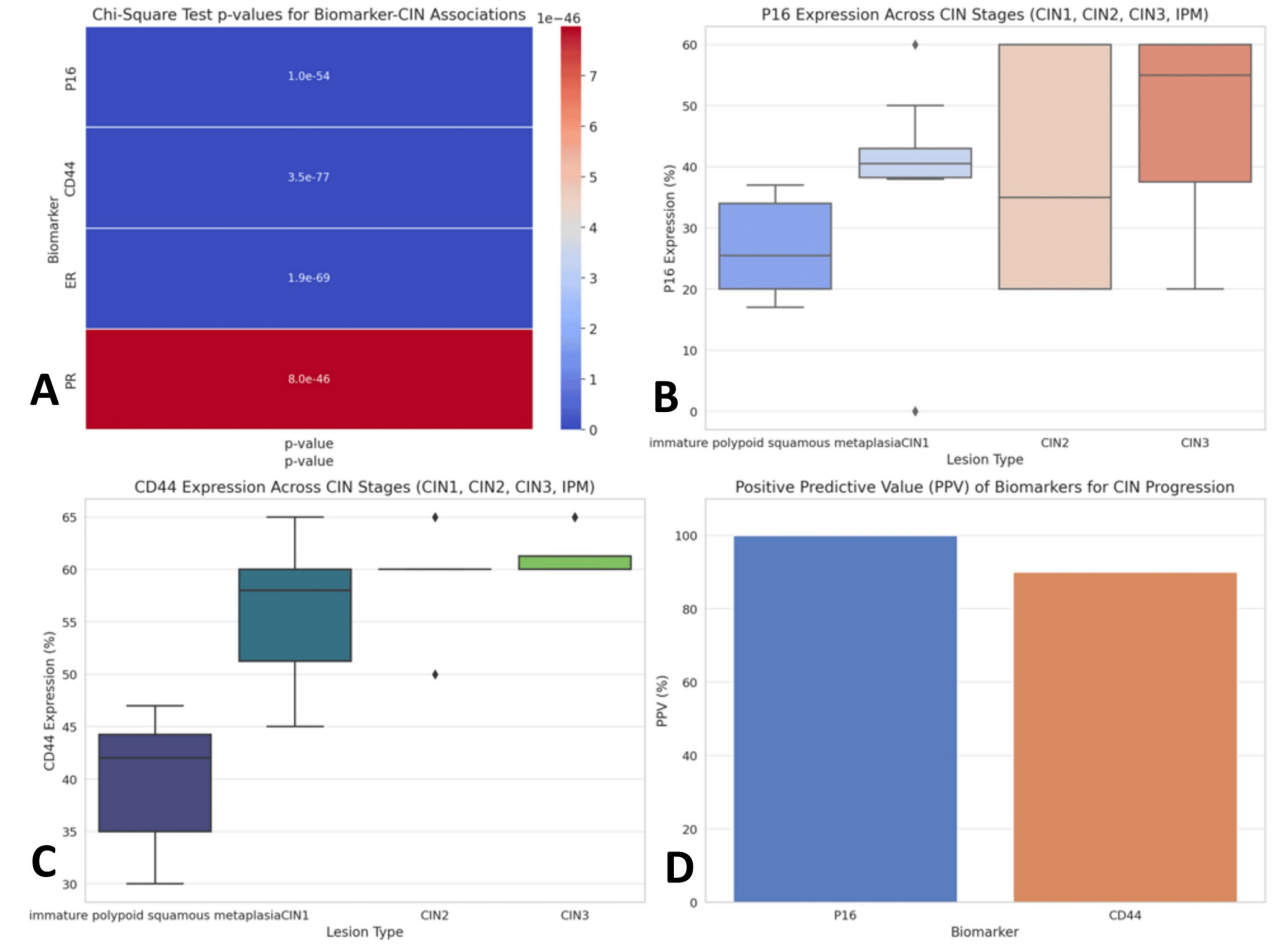
PPV of biomarkers for CIN progression A. Heatmap of chi-square test p-values showing the statistical significance of associations between P16, CD44, ER, and PR with CIN progression. Stronger associations (lower p-values) are highlighted. B. Boxplot of P16 expression across CIN stages showing the increasing P16 expression in higher-grade CIN stages (CIN2/3). C. Boxplot of CD44 expression across CIN stages showing higher CD44 expression in CIN2 and CIN3 compared to IPM. D. Bar plot of PPV for progression showing that P16 (100%) and CD44 (90%) have a strong predictive power for CIN progression. CIN, cervical intraepithelial neoplasia; IPM, immature polypoid squamous metaplasia; PPV, positive predictive value

In contrast, ER and PR expressions did not show a significant association with CIN progression (p = 1.92e-69 for ER, p = 7.98e-46 for PR), indicating that hormonal receptor status may not be a critical factor in the transition from IPM to CIN. For the subgroup of patients who progressed from IPM to CIN, P16 and CD44 were identified as highly predictive biomarkers of progression. P16 showed a 100% positive predictive value (PPV) for progression to CIN2 or CIN3, meaning that all IPM cases with moderate or strong P16 expression advanced to higher-grade CIN.CD44 showed a 90% PPV for progression to CIN, further highlighting its potential as a marker for aggressive cellular behavior. These findings suggest that P16 and CD44 expression levels may serve as critical indicators for identifying IPM cases at risk of progression, particularly to CIN2 and CIN3 (Figure 3).

## Discussion

Our study aimed to evaluate the IHC expression of P16, ER, PR, and CD44 in CIN and IPM, with a particular focus on markers that may have predictive value in the progression from IPM to CIN. Our results show that P16 and CD44 are significantly associated with the severity of CIN and the progression from IPM to CIN, while ER and PR expressions are less reliably associated.

P16 expression was significantly higher in CIN2 and CIN3 cases compared with IPM and reactive controls, which is consistent with existing studies linking P16 upregulation with high-risk HPV infection and the development of dysplasia. Many studies have shown that P16 is an effective adjunctive marker for high-risk HPV infection and induces dysregulation of the retinoblastoma (Rb) protein pathway, which is a critical event in HPV-induced cervical carcinogenesis. For example, da Mata et al. found that P16 is overexpressed in CIN2 and CIN3 and is closely associated with high-risk HPV [10]. In our study, P16 expression was related to the progression of IPM to CIN, with 80% of progressive IPM cases showing moderate or vigorous expression. This is consistent with the findings of Kyrgiou et al., who highlighted P16 as a biomarker for predicting progression from low-grade lesions to high-grade CIN and invasive cervical cancer [11]. Our data reinforce the notion that P16 is a reliable biomarker for identifying high-risk lesions in the cervical epithelium, especially in IPM cases that may be at high risk of progression to CIN2 or CIN3. This finding highlights the utility of P16 as a marker for assessing high-risk HPV-induced cervical carcinogenesis and its potential for use in cervical cancer screening programs.

Our study also demonstrated a significant association between CD44 expression and CIN severity, with intense CD44 staining in CIN2 and CIN3 cases. CD44 is a cell surface glycoprotein involved in cell-cell interactions, adhesion, and migration, and its expression is associated with tumor progression and metastasis in various cancers, including cervical cancer. This is consistent with the findings of de Sousa et al., who found that CD44 overexpression in cervical lesions is associated with increased tumor aggressiveness and the potential for progression to invasive disease [12].

Our study results showed that CD44 was a reliable marker of dysplastic progression, with 70% of progressive IPM cases showing strong expression of CD44. This finding suggests that CD44 may be a marker of EMT, which is associated with tumor progression and metastasis. The strong CD44 expression in CIN2 and CIN3 cases highlights its potential as a prognostic marker for identifying high-risk lesions and predicting invasive cervical cancer.

Our findings are consistent with those of Han et al., who reported that CD44 overexpression in cervical lesions was strongly associated with poor prognosis and an increased risk of progression to invasive disease. The correlation of CD44 expression with IPM progression suggests that CD44 may be an additional biomarker to P16, indicating cervical lesions’ aggressiveness and metastatic potential [13].

The expression of ER and PR in CIN and IPM was less critical in predicting progression compared with P16 and CD44. ER and PR expressions were observed in CIN1, CIN2, and CIN3 cases. This finding aligns with previous studies, which suggested that hormone receptors may not serve as strong indicators of HPV-induced progression in cervical lesions.

Although hormone receptors are involved in the regulation of the cervical epithelium and are implicated in the pathogenesis of cervical cancer, the effects of estrogen and progesterone signaling in HPV-induced lesions may be secondary to the more dominant role of viral oncoproteins, such as HPV E6/E7, in promoting cell dysregulation [14]. In our study, although ER and PR expressions were present in a subset of cases, they did not show a consistent correlation with progression or disease severity, supporting the hypothesis that hormonal signaling is not a major driver in the progression of HPV-associated lesions.

Further research is needed to explore the interaction between hormonal signaling and HPV infection in cervical carcinogenesis. Głowienka-Stodolak et al. found that although hormonal fluctuations may influence the persistence of low-grade lesions, their role in the progression to high-grade dysplasia or invasive cancer may be limited [15].

The observation that P16 and CD44 were more prominently expressed in progressive IPM cases than stable IPM suggests that these markers may help identify IPM lesions with a higher probability of progression to CIN. The PPV of P16 (85%) and CD44 (75%) for progression to CIN2 or CIN3 is significant, as these markers can help clinicians identify high-risk lesions that require closer monitoring and intervention.

These findings are consistent with those of Mehdi et al., who showed that the combined use of P16 and CD44 can predict the likelihood of progression from CIN1 to higher-grade lesions or invasive cancer. Our study's predictive ability of P16 and CD44 supports their potential use in clinical practice for more accurate risk stratification and early intervention in cervical cancer prevention [16].

Although the results of this study provide essential information about the expression of key biomarkers in CIN and IPM, there are several limitations. First, the study's retrospective nature means that a causal relationship between biomarker expression and progression to invasive disease cannot be definitively established. Longitudinal studies that follow patients over time will provide more robust data on the role of these markers in predicting disease progression. Furthermore, the study focused on a limited set of biomarkers, and further investigation of other potential markers of cervical carcinogenesis will contribute to our understanding of the molecular mechanisms underlying cervical cancer progression.

Finally, although P16 and CD44 show promising results as markers of IPM progression, more extensive studies are needed to confirm their clinical utility and refine their role in routine cervical cancer screening and management.

## Conclusions

This study highlights the potential of P16 and CD44 as valuable biomarkers for predicting the progression of IPM to CIN. Our findings suggest that these markers could be used for early detection and risk stratification in cervical cancer prevention, offering a reliable method for identifying high-risk IPM lesions. While estrogen and PR expression were less significant, further research into their role in HPV-driven lesions may provide additional insights. More significant prospective studies are needed to validate these biomarkers in clinical practice and improve cervical cancer screening strategies.
